# Supplementary material for: Burden of Atopic Dermatitis in Adults in Greece: Results from a Nationwide Survey
Source: J Clin Med. 2022 Aug 16;11(16):4777. doi: 10.3390/jcm11164777 (PMC9410403; doi:10.3390/jcm11164777)
Supplement: Supplementary file 1 [file jcm-11-04777-s001.zip › jcm-1839162-supplementary.pdf]

Supplementary A

Survey questionnaire on atopic dermatitis

Introduction

Good morning/good afternoon. My name is ...... and I work in the polling company MRB, which is an independent research company and is currently conducting a study about health issues.

1. **I would first like to ask if you are over 18 years old.**

Yes □

No □

No answer □

If «No» or «No answer» -> THANK THE RESPONDENT AND HANG UP.

If «Yes» -> READ THE STUDY INFORMATION.

We are currently conducting a survey on Atopic Dermatitis and the effects it has on patients.

We would like to assure you that all information you provide to us will remain confidential, without the possibility of identifying specific respondents. Our practices are fully legal and in line with the principles and provisions of the Personal Data Protection Authority and each of your responses will be presented in total, along with the answers of other respondents.

The duration of the overall survey will not exceed the 15 minutes.

2. **Considering what we have told you, would you like to participate in this survey?**

□ Yes (CONTINUE)

□ No (THANK THE RESPONDENT AND HANG UP)

A. DEMOGRAPHICS	
**A1. Participant's Gender**	Male □ Female □
**A2. Participant's Age** (MARK THE EXACT AGE IN YEARS) _____	□ 18 – 29<br>□ 30 – 39<br>□ 40 – 49<br>□ 50 – 59<br>□ 60 – 69<br>□ 70+
**A3. Based on the number of inhabitants, in which of the following categories does the household belongs to?**	□ Athens<br>□ Thessaloniki<br>□ Urban area (over 10,000 inhabitants)<br>□ Semi-urban area (2,000 – 10,000 inhabitants)<br>□ Rural area (up to 2,000 inhabitants)
**A4. Weight**	…………………. (in kilograms)
**A5. Height**	…………………. (in meters)
**A6. Marital status**	□ Unmarried<br>□ Married / in cohabitation<br>□ Separated<br>□ Widow/er
**A7a. Professional status**	□ Self-employed/freelancer<br>□ Employee<br>□ Unemployed<br>□ Retired<br>□ Student<br>□ Household<br>□ other
**A7b. If you are an employee or a freelancer please indicate if you are doing any of the following professions?**	□ Hairdresser/beautician<br>□ Food Industry Worker<br>□ health/dentist/veterinarian<br>□ laboratory worker/laboratory technician<br>□ farmer, gardener, florist<br>□ cleaning worker<br>□ painter, painter, décor<br>□ repairs of motor vehicles<br>□ construction worker<br>□ typographer
**A8. Level of education**	□ Primary school<br>□ Lower Secondary school<br>□ Upper Secondary school

A. DEMOGRAPHICS	
	□ Bachelor’s degree<br>□ Master’s /Doctoral degree
**A9. Have you been diagnosed by your doctor or are you being treated for the following diseases?**<br>**Select as many as apply.**	□ asthma<br>□ chronic obstructive pulmonary disease<br>□ allergies (Allergic rhinitis, conjunctivitis, food allergies)<br>□ gastrointestinal problems<br>□ seasonal rhinitis<br>□ diabetes<br>□ hypertension<br>□ heart failure<br>□ Other: ………………………………… (fill in which)
**A10. Do you have a family history of atopic dermatitis, rhinitis, or asthma?**	□ Yes<br>□ No<br>□ No Answer

B. HISTORY OF ATOPIC DERMATITIS	
**B1. In the last 12 months, have you suffered from a generally dry skin?**	Yes □ No □ No answer □
**B2. Do you suffer or have you suffered from asthma (bouts of wheezing with coughing)?**	Yes □ No □ No answer □
**B3a. Do you have a diagnosis of atopic dermatitis or eczema by your doctor?**	Yes □ No □ No answer □
**B3b. If yes, which was your age at the 1st diagnosis.** (If the respondent answered "No" at B3a, do not ask this. Go to B4a)	…………………
**B4a. Have you ever had an itchy rash which was coming and going for at least 6 months?**	Yes □ No □ No answer □
Regardless of the answers at B1 and B2:<br><br>If B4a = "No", and B3a (green)= "No", thank the respondent and hang up.<br><br>If B4a = "No", and B3a (green)= "Yes", go to section C.<br><br>If B4a = "Yes", go to B4b.	
**B4b. When this itchy rash first occur, your age was under 2 years?**	Yes □ No □ No answer □
**B5. Has this itchy rash at any time affected any of the following places: the folds of the elbows, behind the knees, in front of the ankles, under the buttocks or around the neck, ears, or eyes?**	Yes □ No □ No answer □
**B6. Have you had this itchy rash in the past 12 months?**	Yes □ No □ No answer □
If B6 = "No", and B3a (green)= "No", thank the respondent and hang up.<br><br>If B6 = "No", and B3a (green)= "Yes", go to section C.	
If B6 = "Yes" (blue) and the answers at<br><br>B1, B2, B4b, B5 (orange) have 0, 1 or 2 "Yes", thank the respondent and hang up.<br><br>**[We ignore this if B3a (green) = "Yes"]**	

<table>
<tr><th colspan="2">C. SMOKING HABITS</th></tr>
<tr><td>C1. In which of the following categories, regarding your smoking habits, you belong?</td><td>□ Current Smoker
□ Former smoker
□ Non-smoker</td></tr>
<tr><td colspan="2">«Note: if the answer was "Current" or "Former Smoker" go to question C2, if the answer was "Non-smoker" go to section D.</td></tr>
<tr><td>C2. What tobacco products do you use?</td><td>□ Cigarettes
□ electronic cigarettes (nicotine reception via steam)
□ heated tobacco products, such as IQOS</td></tr>
</table>

D. Patient Oriented Eczema Measure	
**D1. During the last week, how many days have you felt itchy because of your eczema?**	□ None<br>□ 1-2 days<br>□ 3-4 days<br>□ 5-6 days<br>□ Every day
**D2. During the last week, how many days has your sleep been disturbed because of your eczema?**	□ None<br>□ 1-2 days<br>□ 3-4 days<br>□ 5-6 days<br>□ Every day
**D3. During the last week, how many days has your skin been bleeding because of your eczema?**	□ None<br>□ 1-2 days<br>□ 3-4 days<br>□ 5-6 days<br>□ Every day
**D4. During the last week, how many days has your skin been secreted clear liquid because of your eczema?**	□ None<br>□ 1-2 days<br>□ 3-4 days<br>□ 5-6 days<br>□ Every day
**D5. During the last week, how many days has your skin been cracked because of your eczema?**	□ None<br>□ 1-2 days<br>□ 3-4 days<br>□ 5-6 days<br>□ Every day
**D6. During the last week, how many days has your skin been peeled off because of your eczema?**	□ None<br>□ 1-2 days<br>□ 3-4 days<br>□ 5-6 days<br>□ Every day
**D7. During the last week, how many days have you felt your skin dry or rough because of your eczema?**	□ None<br>□ 1-2 days<br>□ 3-4 days<br>□ 5-6 days<br>□ Every day

E. Quality of life (DLQI)	
**E1. Over the last week, how itchy, sore, painful or stinging has your skin been?**	☐ Very much<br>☐ A lot<br>☐ A little<br>☐ Not at all
**E2. Over the last week, how embarrassed or self conscious have you been because of your skin?**	☐ Very much<br>☐ A lot<br>☐ A little<br>☐ Not at all
**E3. Over the last week, how much has your skin interfered with you going shopping or looking after your home or garden?**	☐ Very much<br>☐ A lot<br>☐ A little<br>☐ Not at all<br>☐ Not relevant
**E4. Over the last week, how much has your skin influenced the clothes you wear?**	☐ Very much<br>☐ A lot<br>☐ A little<br>☐ Not at all<br>☐ Not relevant
**E5. Over the last week, how much has your skin affected any social or leisure activities?**	☐ Very much<br>☐ A lot<br>☐ A little<br>☐ Not at all<br>☐ Not relevant
**E6. Over the last week, how much has your skin made it difficult for you to do any sport?**	☐ Very much<br>☐ A lot<br>☐ A little<br>☐ Not at all<br>☐ Not relevant
**E7a. Over the last week, has your skin prevented you from working or studying?**	☐ Yes<br>☐ No<br>☐ Not relevant
**E7b. If "No", over the last week how much has your skin been aproblem at work or studying?**	☐ Very much<br>☐ A lot<br>☐ A little<br>☐ Not at all<br>☐ Not relevant
**E8. Over the last week, how much has your skin created problems with your partner or any of your close friends or relatives?**	☐ Very much<br>☐ A lot<br>☐ A little<br>☐ Not at all<br>☐ Not relevant

E. Quality of life (DLQI)	
**E9. Over the last week, how much has your skin caused any sexual difficulties?**	◻ Very much<br>◻ A lot<br>◻ A little<br>◻ Not at all<br>◻ Not relevant
**E10. Over the last week, how much of a problem has the treatment for your skin been, for example by making your home messy, or by taking up time?**	◻ Very much<br>◻ A lot<br>◻ A little<br>◻ Not at all<br>◻ Not relevant

F. Work Productivity and Activity Impairment Questionnaire

The following questions ask about the effect of your health problems on your ability to work and perform regular activities.

**F1. Are you currently employed (working for pay)?**	No □ Yes □

If NO, check "NO" and skip to question F6.

The next questions are about the past seven days, not including today.

**F2. During the past seven days, how many hours did you miss from work because of atopic dermatitis?** Include hours you missed on sick days, times you went in late, left early, etc., because of atopic dermatitis. Do not include time you missed to participate in this study.	……… hours
**F3. During the past seven days, how many hours did you miss from work because of any other reason, such as vacation, holidays, time off to participate in this study?**	……… hours
**F4. During the past seven days, how many hours did you actually work?**	……… hours

(If "0", skip to question F6.)

F5. During the past seven days, how much did atopic dermatitis affect your productivity while you were working?

Think about days you were limited in the amount or kind of work you could do, days you accomplished less than you would like, or days you could not do your work as carefully as usual. If atopic dermatitis affected your work only a little, choose a low number. Choose a high number if atopic dermatitis affected your work a great deal.

Consider only how much atopic dermatitis affected productivity while you were working.

0	1	2	3	4	5	6	7	8	9

F6. During the past seven days, how much did your atopic dermatitis affect your ability to do your regular daily activities, other than work at a job?

By regular activities, we mean the usual activities you do, such as work around the house, shopping, childcare, exercising, studying, etc. Think about times you were limited in the amount or kind of activities you could do and times you accomplished less than you would like. If atopic dermatitis affected your activities only a little,

F. Work Productivity and Activity Impairment Questionnaire
The following questions ask about the effect of your health problems on your ability to work and perform regular activities.
choose a low number. Choose a high number if atopic dermatitis affected your activities a great deal. **Consider only how much atopic dermatitis affected your ability to do your regular daily activities, other than work at a job.**

0	1	2	3	4	5	6	7	8	9

G. Impact on sleep and social life	
**G1. In the last 12 months, how much is your sleep quality generally affected due to AD?**	◻ none at all<br>◻ a little<br>◻ moderate<br>◻ much<br>◻ very much
**G2. In the last 12 months, how often has AD prevented you from responding to your social life?**	◻ never<br>◻ little<br>◻ somewhat<br>◻ much<br>◻ a great deal

**H. Resource use**	
**H1. In the last 12 months how many times did you have to visit your doctor's private office due to your atopic dermatitis?**	……… (times)
**H2. In the last 12 months have you received any treatment regarding your atopic dermatitis?**	Yes □ No □
If "No", thank the respondent and hang up	
**H3a.** *If the respondent has answered yes:*<br>**What kind of treatment are you currently receiving or received in the last 12 months?**	□ Management with specific nutrition<br><br>□ Topical treatment with cream or ointments with moisturizing or anti-inflammatory action, such as corticosteroid or calcineurin inhibitors,<br><br>□ Phototherapy<br><br>□ Oral systemic treatment as cortisone, cyclosporine, antihistamines<br><br>□ Antibiotics, as cephalosporin<br><br>□ Immunotherapy, as azathioprine, mycophenolate mofetil, methotrexate<br><br>□ Biologic agents, as dupilumab (dupixent), rituximab (MabThera), mepolizumab (Nucala), omalizumab (Xolair), ustekinumab (Stelara)
**H3b.** *If the respondent has answered yes but does not know or remember the type of treatment:*<br>**The treatment you are receiving is:**	□ cream or ointment<br><br>□ oral treatment, pills or syrup<br><br>□ treatment in injectable form

End of interview.

Supplementary B

Table S1 DLQI total score and subscales by study cohort and AD severity (last 7 days).

	UKWP				Expert diagnosis			
	Overall N=69	Clear or almost clear skin/mild eczema N=32	Moderate to very severe eczema N=36	p	Overall N=349	Clear or almost clear skin/mild eczema N=252	Moderate to very severe eczema N=88	p
**DLQI score**, mean (95% CI)	10.92 (9.24, 12.59)	9.42 (6.99, 11.85)	12.66 (10.48, 14.84)	0.052	5.29 (4.66, 5.93)	3.70 (3.11, 4.30)	10.07 (8.71, 11.43)	<0.001
**DLQI subscales**, mean (95% CI)								
Symptoms and feelings	3.50 (3.16, 3.83)	3.43 (2.96, 3.90)	3.64 (3.18, 4.10)	0.517	2.23 (2.04, 2.41)	1.76 (1.56, 1.96)	3.52 (3.19, 3.85)	<0.001
Daily activities	2.32 (1.88, 2.76)	1.99 (1.34, 2.65)	2.68 (2.10, 3.26)	0.122	1.03 (0.87, 1.19)	0.66 (0.51, 0.82)	2.05 (1.68, 2.42)	<0.001
Leisure	2.06 (1.64, 2.48)	1.64 (1.02, 2.27)	2.50 (1.97, 3.02)	0.042	0.90 (0.75, 1.06)	0.52 (0.38, 0.67)	1.94 (1.58, 2.29)	<0.001
Work and school	0.38 (0.22, 0.54)	0.22 (0.04, 0.39)	0.55 (0.30, 0.80)	0.032	0.24 (0.18, 0.30)	0.14 (0.09, 0.19)	0.53 (0.38, 0.69)	<0.001
Personal relationships	1.86 (1.40, 2.33)	1.52 (0.85, 2.19)	2.24 (1.60, 2.89)	0.124	0.72 (0.57, 0.86)	0.44 (0.30, 0.57)	1.45 (1.09, 1.80)	<0.001
Treatment	0.93 (0.70, 1.15)	0.62 (0.31, 0.93)	1.23 (0.92, 1.53)	0.007	0.39 (0.31, 0.46)	0.18 (0.12, 0.25)	0.93 (0.75, 1.11)	<0.001

UKWP: UK Working Party; AD: atopic dermatitis; DLQI: dermatology life quality index.

All p-values displayed were derived by a two-sample t-test.

Table S2 Univariate and multiple models for the impact on QoL by study cohort and AD severity.

	UKWP				Expert diagnosis			
	Univariate N=67		Multiple N=66		Univariate N=324		Multiple N=302	
**Impact on DLQI**	**OR (95% CI)**	**p**	**OR (95% CI)**	**p**	**OR (95% CI)**	**p**	**OR (95% CI)**	**p**
**Gender (females vs. males)**	0.39 (0.12, 1.27)	0.117	0.28 (0.06, 1.33)	0.110	1.20 (0.74, 1.94)	0.457	1.49 (0.81, 2.75)	0.199
**Age, decades**	0.79 (0.53, 1.15)	0.220	0.68 (0.40, 1.13)	0.137	0.74 (0.62, 0.87)	<0.001	0.78 (0.62, 0.99)	0.042
**BMI (vs. Underweight/Normal)**		0.143‡		0.209‡		0.592‡		0.574‡
Overweight	2.79 (0.86, 9.08)	0.088	3.71 (0.68, 20.4)	0.131	1.03 (0.62, 1.72)	0.903	1.30 (0.67, 2.53)	0.442
Obese	3.64 (0.62, 21.3)	0.152	4.48 (0.54, 37.2)	0.165	1.39 (0.73, 2.65)	0.319	1.52 (0.65, 3.55)	0.336
**Time since AD diagnosis, years**	NA				0.98 (0.96, 1.00)	0.058	0.99 (0.96, 1.01)	0.316
**Comorbidities (each vs. no)**								
Atopy (asthma, allergies, rhinitis)	0.70 (0.21, 2.31)	0.553			2.37 (1.47, 3.81)	<0.001	1.84 (1.02, 3.32)	0.044
COPD	--				1.86 (0.36, 9.53)	0.458		
GI problems	0.38 (0.11, 1.32)	0.128			2.11 (1.15, 3.86)	0.016	2.69 (1.26, 5.76)	0.011
Diabetes	0.68 (0.12, 3.88)	0.667			0.89 (0.32, 2.47)	0.826		
Hypertension	0.60 (0.13, 2.71)	0.506			0.57 (0.29, 1.15)	0.116		
Heart failure	0.72 (0.06, 8.11)	0.794			2.33 (0.52, 10.5)	0.269		
**Smoking (vs. Smoker)**		0.041‡		0.092‡		0.017‡		0.091‡
Former smoker	0.37 (0.10, 1.40)	0.143	0.30 (0.06, 1.44)	0.133	0.73 (0.42, 1.30)	0.289	0.88 (0.44, 1.75)	0.720
No smoker	0.16 (0.4, 0.67)	0.012	0.14 (0.02, 0.87)	0.035	0.44 (0.25, 0.78)	0.005	0.46 (0.22, 0.95)	0.035
**Disease severity (POEM) (**Moderate to very severe eczema vs. Clear/ clear skin/ Mild eczema)	4.27 (1.30, 13.95)	0.016	5.37 (1.28, 22.6)	0.022	8.82 (4.91, 15.8)	<0.001	7.89 (4.18, 14.9)	<0.001

UKWP: UK Working Party; AD: atopic dermatitis; DLQI: dermatology life quality index; OR: odds ratio; CI: confidence interval; NA: not applicable.

Logistic regression models were applied with dependent variable Y = {1=’*moderate to extremely large effect’, 0=’no effect to small effect’*} based on DLQI score

‡Wald test.

Table S3 Univariate and multiple models for the impact on sleep by study cohort and AD severity

	UKWP				Expert diagnosis			
	Univariate N=68		Multiple N=67		Univariate N=345		Multiple N=322	
**Impact on sleep**	**OR (95% CI)**	**p**	**OR (95% CI)**	**p**	**OR (95% CI)**	**p**	**OR (95% CI)**	**p**
**Gender (females vs. males)**	0.81 (0.30, 2.19)	0.673	0.98 (0.29, 3.31)	0.975	0.94 (0.56, 1.57)	0.806	1.08 (0.59, 1.98)	0.801
**Age, decades**	0.85 (0.59, 1.23)	0.387	0.83 (0.53, 1.30)	0.424	0.76 (0.64, 0.92)	0.004	0.80 (0.64, 0.99)	0.043
**BMI (vs.** Underweight/Normal)		0.045‡		0.020‡		0.169‡		0.106‡
Overweight	2.47 (0.83, 7.34)	0.102	3.34 (0.88, 12.7)	0.077	0.72 (0.40, 1.28)	0.258	0.91 (0.45, 1.83)	0.783
Obese	8.06 (1.37, 47.6)	0.021	13.6 (1.91, 96.4)	0.009	1.42 (0.74, 2.74)	0.295	2.18 (0.94, 5.05)	0.068
**Time since AD diagnosis, years**	NA				0.99 (0.97, 1.01)	0.387		
**Comorbidities (each vs. no)**								
Atopy (asthma, allergies, rhinitis)	0.79 (0.28, 2.29)	0.668			3.02 (1.77, 5.14)	<0.001	2.69 (1.44, 5.03)	0.002
COPD	--				3.34 (0.80, 14.0)	0.099	0.98 (0.17, 5.65)	0.983
GI problems	0.40 (0.12, 1.38)	0.149			1.56 (0.83, 2.92)	0.166		
Diabetes	0.79 (0.15, 4.24)	0.784			2.06 (0.82, 5.17)	0.123		
Hypertension	0.27 (0.05, 1.39)	0.117			0.73 (0.35, 1.54)	0.414		
Heart failure	0.44 (0.04, 5.17)	0.514			1.90 (0.45, 8.01)	0.382		
**Smoking (vs. Smoker)**		0.214‡				0.059		0.603‡
Former smoker	0.99 (0.31, 3.17)	0.982			0.62 (0.33, 1.14)	0.123	0.85 (0.41, 1.74)	0.656
No smoker	0.33 (0.09, 1.21)	0.095			0.50 (0.28, 0.91)	0.022	0.69 (0.33, 1.43)	0.315
**Disease severity (POEM) (**Moderate to very severe eczema vs. Clear/ clear skin/ Mild eczema)	3.69 (1.31, 10.4)	0.013	5.31 (1.59, 17.7)	0.007	7.48 (4.27, 13.1)	<0.001	5.61 (3.07, 10.3)	<0.001

UKWP: UK Working Party; AD: atopic dermatitis; DLQI: dermatology life quality index; OR: odds ratio; CI: confidence interval; NA: not applicable.

Logistic regression models were applied with dependent variable Y = {1= *'moderate to very large effect on sleep', 0='none to small effect on sleep'*}

‡Wald test.

Table S4 Univariate and multiple models for the impact on social life by study cohort and AD severity

	UKWP				Expert diagnosis			
	Univariate N=68	p	Multiple N=67	p	Univariate N=345	p	Multiple N=315	p
**Impact on social life**	**OR (95% CI)**		**OR (95% CI)**		**OR (95% CI)**		**OR (95% CI)**	
**Gender (females vs. males)**	0.71 (0.26, 1.92)	0.494	0.74 (0.22, 2.52)	0.631	0.85 (0.50, 1.44)	0.540	1.03 (0.52, 2.03)	0.941
**Age, decades**	0.77 (0.52, 1.14)	0.189	0.80 (0.51, 1.27)	0.347	0.74 (0.61, 0.90)	0.002	0.73 (0.56, 0.96)	0.026
**BMI (vs.** Underweight/Normal)		0.153‡		0.065‡		0.097‡		0.391‡
Overweight	1.58 (0.54, 4.67)	0.404	1.86 (0.50, 6.96)	0.358	0.88 (0.48, 1.61)	0.687	1.35 (0.62, 2.90)	0.449
Obese	4.78 (0.98, 23.3)	0.053	8.76 (1.41, 54.4)	0.020	1.88 (0.96, 3.69)	0.067	1.94 (1.74, 5.12)	0.178
**Time since AD diagnosis, years**	NA				0.97 (0.95, 0.99)	0.015		
**Comorbidities (each vs. no)**								
Atopy (asthma, allergies, rhinitis)	0.56 (0.19, 1.61)	0.278			2.32 (1.35, 3.98)	0.002	1.82 (0.90, 3.66)	0.094
COPD	--				3.73 (0.89, 15.7)	0.072		
GI problems	1.08 (0.33, 3.54)	0.894			1.37 (0.71, 2.65)	0.353		
Diabetes	1.93 (0.35, 10.8)	0.453			2.85 (1.14, 7.10)	0.024	2.98 (0.83, 10.6)	0.093
Hypertension	0.61 (0.13, 2.78)	0.522			0.87 (0.41, 1.84)	0.719		
Heart failure	1.94 (0.18, 21.5)	0.589			3.42 (0.84, 13.9)	0.085		
**Smoking (vs. Smoker)**		0.173‡				0.011‡		0.207‡
Former smoker	0.71 (0.22, 2.25)	0.561			0.55 (0.29, 1.04)	0.064	0.71 (0.33, 1.56)	0.395
No smoker	0.27 (0.07, 1.06)	0.061			0.39 (0.21, 0.74)	0.004	0.47 (0.21, 1.09)	0.078
**Disease severity (POEM) (**Moderate to very severe eczema vs. Clear/ clear skin/ Mild eczema)	4.79 (1.66, 13.9)	0.004	6.61 (1.94, 22.6)	0.003	9.87 (5.47, 17.8)	<0.001	7.07 (3.64, 13.7)	<0.001

UKWP: UK Working Party; AD: atopic dermatitis; DLQI: dermatology life quality index; OR: odds ratio; CI: confidence interval; NA: not applicable.

Logistic regression models were applied with dependent variable Y = {1= *'somewhat to a great deal effect on social life, 0='never to little effect on social life'*}

‡Wald test.

Table S5 Univariate and multiple models for the impact on overall work impairments by study cohort and AD severity.

	UKWP				Expert diagnosis			
	Univariate N=43		Multiple N=42		Univariate N=171		Multiple N=164	
**Impact on overall work impairments**	**Exp(b) (95% CI)**	**p**	**Exp(b) (95% CI)**	**p**	**Exp(b) (95% CI)**	**p**	**Exp(b) (95% CI)**	**p**
**Gender (females vs. males)**	1.03 (0.65, 1.61)	0.914	1.09 (0.74, 1.61)	0.660	1.05 (0.67, 1.62)	0.839	1.41 (0.90, 2.23)	0.136
**Age, decades**	0.85 (0.67, 1.09)	0.194	0.92 (0.74, 1.15)	0.486	0.80 (0.65, 0.98)	0.033	0.89 (0.96, 1.00)	0.119
**BMI (vs.** Underweight/Normal)		0.002‡		<0.001‡		0.003‡		0.582‡
Overweight	1.24 (0.75, 2.05)	0.406	1.12 (0.68, 1.85)	0.657	1.25 (0.75, 2.09)	0.395	1.12 (0.73, 1.71)	0.604
Obese	2.28 (1.38, 3.77)	0.001	2.32 (1.49, 3.63)	<0.001	2.25 (1.39, 3.63)	0.001	1.30 (0.79, 2.14)	0.298
**Time since AD diagnosis, years**	NA				0.98 (0.96, 1.00)	0.104		
**Comorbidities (each vs. no)**								
Atopy (asthma, allergies, rhinitis)	0.74 (0.48, 1.14)	0.172			1.72 (1.06, 2.78)	0.027		
COPD	2.25 (1.11, 4.58)	0.025	1.90 (0.91, 3.98)	0.089	3.79 (2.29, 2.26)	<0.001		
GI problems	1.36 (0.85, 2.16)	0.199			1.95 (1.29, 2.94)	0.001	1.45 (0.93, 2.26)	0.102
Diabetes	1.98 (1.23, 3.18)	0.005			2.28 (1.36, 3.80)	0.002		
Hypertension	1.40 (0.80, 2.45)	0.245			1.85 (1.15, 2.96)	0.011	1.86 (1.22, 2.83)	0.004
Heart failure	2.34 (1.44, 3.81)	0.001			5.13 (3.46, 7.59)	<0.001		
**Smoking (vs. Smoker)**		0.401‡				0.092‡		0.487‡
Former smoker	0.67 (0.38, 1.20)	0.178			0.71 (0.44, 1.14)	0.156	0.98 (0.63, 1.53)	0.929
No smoker	0.89 (0.50, 1.56)	0.680			0.53 (0.28, 1.00)	0.052	0.71 (0.39, 1.29)	0.267
**Disease severity (POEM) (**Moderate to very severe eczema vs. Clear/ clear skin/ Mild eczema)	1.49 (0.96, 2.31)	0.078	1.51 (1.02, 2.225)	0.041	2.62 (1.74, 3.94)	<0.001	2.20 (1.47, 3.30)	<0.001

UKWP: UK Working Party; AD: atopic dermatitis; DLQI: dermatology life quality index; OR: odds ratio; CI: confidence interval; NA: not applicable.

Generalized linear models (GLM) with a log link function under the gaussian distribution were fitted.

‡Wald test.
